# Infantile Leucorrhœa

**Published:** 1883-07

**Authors:** 


### Infantile Leucorrhœa.

Clinical lecture by Prof. T. Gaillard Thomas:

Gentlemen—The little girl, nine years old, whom I first bring before you, is suffering from a very profuse leucorrhœa, which, her mother informs me, she has been unable to cure by any of the remedies which she has employed, and which has now lasted for two months. I, of course, made a vaginal examination, and, on separating the labia, I found that the whole vulva was about the color of red flannel, and bathed with a copious leucorrhœal discharge. The meatus urinarius was also seen to be in the same condition, and urethritis has, no doubt, been set up by the spreading of the irritation. If it had been necessary, I could have introduced a small glass speculum into the vagina; but this was not required to make a diagnosis, as I saw exactly what was the matter without resorting to this.

Not infrequently mothers will bring their little girls to you in this condition, and they will sometimes be in a state of great agitation, because they are afraid the trouble has been the result of injury done the children. There is ordinarily no reason whatever to suspect anything of the kind, and you can at once quiet the anxious mother's mind. The affection is a perfectly simple one, and is perfectly curable also. What is it, then? It is generally known as infantile leucorrhœa; but infantile vaginitis would be a better term for it.

Now as to its causes. One of the most frequent of these is neglect of hygienic precautions. There is generally no intentional neglect on the part of the mother or nurse; but, on account of the undeveloped condition of the part, an accumulation of hardened secretion sometimes collects in the same way as that which not infrequently gives rise to balanitis in the male child. Another common cause is the depreciated condition of the child's system, such as that due to spanæmia, in which all the mucous membrances are apt to become more or less affected. Thus, there is often gastric and intestinal, as well as nasal catarrh. A third cause that may be mentioned is reflex influence from the rectum. The cause of the irritation in the rectum is usually ascarides, and an afflux of blood to the part is caused by the itching and irritation.

In some instances, the ascarides, by getting into the vagina itself, are the direct cause of the trouble. The prognosis of this affection is, that it can be cured at once if it is properly treated.

In the treatment, the first thing to do is to see if there are any worms present, and if so (or there is any reason to suspect that such is the case), use an injection of warm salt water, as this form of ascaris (the *ascaris vermicularis*), as well as others, is unfavorably affected by salt. The next thing to do is to get the child's general system in the best condition possible by appropriate food, iron, vegetable tonics, and the hypophosphites. It is better to depend on nourishing diet, however, than on medicinal agents. If, after the worms have been gotten rid of, the vaginal irritation and discharge should continue, or if no worms should be found to be present, local treatment will be required. The vagina should be thoroughly washed out by means of a syringe provided with a small nozzle, which ought to be well oiled before being introduced. In order that the canal may be perfectly cleansed, the child should be placed upon the back. In some cases the mere removal of the accumulated secretion, which is a constant source of irritation, is all that is necessary; but if the trouble has gone on for some time, this may not be sufficient. Something further is then needed, and one of the best applications to use is the old-fashioned black wash (calomel and lime-water) in the strength of one ounce to a pint of water. Before using this (which should be done twice a day) an injection of simple warm water should be made. I have never yet seen a case of infantile leucorrhœa that could not be cured by such treatment as this; so that there is no necessity of resorting to astringents and nitrate of silver, which may perhaps do harm. If it is adopted here, I have no doubt that in less than two weeks this child will be entirely well.

But there is one mistake which is apt to be made by the physician in these cases, on account of which a much longer time may be required for a case than is at all necessary, and that is, the failure on his part to show the mother or nurse how to introduce the nozzle of the syringe properly. Mothers, unless they are especially instructed in regard to this point, never carry the nozzle more than an eighth of an inch up into the vagina, and as it is above this that the degenerating pus is found, there will be no improvement, simply because the injections fail to reach the real source of trouble. It is not enough even to show the mother how to use the syringe, but you should also watch her do it, and see that the upper part of the vagina is reached. In a child of this age, the rectal tube of a Davidson syringe should be employed.—*Medical and Surgical Reporter.*

